# Single-Incision Laparoscopic Cholecystectomy Using the Marionette Transumbilical Approach Is Safe and Efficient with Careful Patient Selection: A Comparative Analysis with Conventional Multiport Laparoscopic Cholecystectomy

**DOI:** 10.1055/s-0042-1759772

**Published:** 2023-04-10

**Authors:** Amir H. Sohail, Jeffrey Silverstein, Hazim Hakmi, Tulio Brasileiro Silva Pacheco, Yousaf B. Hadi, Manesh Kumar Gangwani, Muhammad Aziz, Hana Ajouz, David Shin

**Affiliations:** 1 Department of Surgery, NYU Langone Hospital–Long Island, Mineola, New York; 2 Department of Medicine, West Virginia University, Morgantown, West Virginia; 3 Department of Medicine, The University of Toledo, Toledo, Ohio

**Keywords:** single-incision laparoscopic, cholecystectomy, marionette technique

## Abstract

**Objectives** The “marionette technique” for transumbilical laparoscopic cholecystectomy (m-TLC) offers improved cosmesis and possibly shorter postoperative recovery for patient undergoing laparoscopic cholecystectomy versus the four-port conventional laparoscopic cholecystectomy (CLC). We compared the outcomes of m-TLC and CLC at a tertiary care facility in New York.

**Methods** A retrospective chart review was conducted and data on patients who underwent m-TLC and CLC were retrieved. Hospital length of stay (LOS), operative time, and complications were compared between the two groups using linear and logistic regression, as appropriate.

**Results** M-TLC group patients were significantly younger, predominantly females with lower body mass index. They were less likely to have previous abdominal surgery and more likely to have noninflammatory pathology ( *p* < 0.05 for all). Nonadjusted LOS (1 vs. 3 days, *p* -value < 0.0001) and operative time (50 vs. 56 minutes, *p* -value = 0.007) were significantly lower among patients who underwent m-TLC; however, there was no significant difference on multivariate analysis. In multivariate analysis, there was no difference in the overall complication rate (odds ratio: 1.63; 95% confidence interval 0.02–2.39).

**Conclusion** With careful patient selection, m-TLC offers better cosmesis with comparable safety outcomes.

**Level of evidence** Level III.

The National Institutes of Health consensus statement first recognized laparoscopic cholecystectomy as “the treatment of choice for many patients” in 1992. 1 Advancement in laparoscopic technology and improved laparoscopic expertise has since resulted in the three- or four-port conventional laparoscopic cholecystectomy (CLC) becoming the standard of care for symptomatic cholelithiasis. Today, ∼460,000 laparoscopic cholecystectomies are performed in the United States annually. Laparoscopic approach is preferred over open cholecystectomy for several reasons, including superior outcomes in terms of safety, cosmesis, recovery time and hospital length of stay (LOS), and cost-effectiveness. 2 3

Newer innovative techniques that attempt to further push boundaries and improve outcomes include the natural orifice transluminal endoscopic surgery, single-incision laparoscopic cholecystectomy (SILC), and robot-assisted SILC. 4 SILC is cost-effective, reduces postoperative pain, and improves cosmetic results and patient satisfaction. However, its major downside, as evidenced in high-quality randomized controlled trials, is a higher incidence of incisional hernias and adverse events. 5 6 7 Interestingly, the “marionette technique” for transumbilical laparoscopic cholecystectomy (m-TLC), first described in 2011 by Kuroki et al, 8 offers the same benefits as SILC but may have improved safety profile. 9 10 11

We present a retrospective comparative analysis of a single surgeon's experience comprising 339 consecutive laparoscopic cholecystectomies; 259 patients underwent four-port CLC, while 80 patients underwent m-TLC.

## Materials and Methods

### Patient Selection and Data Collection

Patients aged ≥18 years presenting to a single minimally invasive surgeon at a tertiary care facility in New York were evaluated for m-TLC or CLC. Patients were selected to undergo m-TLC based on surgeon preference that was predicated upon several factors, including age, sex, acuity and severity of disease, and body habitus. Need for an intraoperative cholangiogram (IOC) or extensive lysis of adhesions, pregnancy, morbid obesity, among other factors, precluded an m-TLC.

Electronic medical records were queried to obtain data. A total of 339 patients underwent laparoscopic cholecystectomy: 259 CLC and 80 m-TLC. Patients with both cholecystitis (acute or chronic) and biliary colic were included in analyses. Information on demographics (age, sex), operative indication, body mass index (BMI), prior abdominal surgery, American Society of Anesthesiologists (ASA) physical status classification, operative time (defined as the time between skin incision and closure of last wound), estimated blood loss, intra- and postoperative complications, conversion to open procedure, conversion from m-TLC to CLC, IOC, and hospital LOS were retrieved.

### Operative Technique

The patient is placed in supine position, and prepped and draped in the usual sterile fashion. Two large caliber braided sutures (#1 Vicryl on a CTX needle) are prepared: one needle is completely straightened out with a small loop at the end of the suture, while the other maintains some curvature.

Vertical 11- and 5-mm infra- and supraumbilical incisions, respectively, are made. After abdominal insufflation using a Veress needle, a 5-mm trocar is introduced in the inferior incision. A 5-mm 30-degree laparoscope is introduced, the abdominal cavity is inspected, and another 5-mm trocar is placed into the superior incision. The straightened needle with the loop at the end of the suture is introduced into the abdomen via the trocar, and is passed through the gallbladder fundus, followed by the loop and the anterior abdominal wall just below costal margin. The needle is removed, and the suture is clamped.

The slightly curved needle is introduced into the abdomen through the abdominal wall to the right of the falciform ligament. Using a laparoscopic needle driver, it is passed through the gallbladder neck thrice and then out through the lateral abdominal wall. The needle is cut, and the ends of the suture are used to retract the gallbladder neck medially and laterally.

**Fig. 1:**
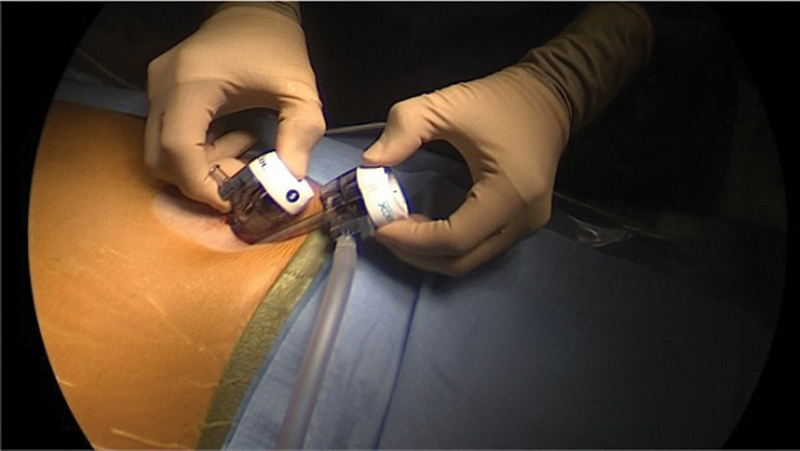
Umbilical ports in single-incision laparoscopic cholecystectomy.

**Fig. 2:**
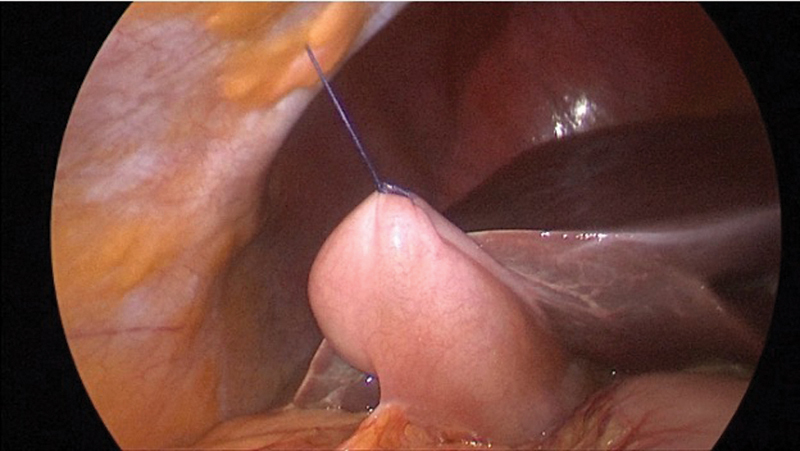
Needle retraction of gallbladder fundus in single-incision laparoscopic cholecystectomy.

**Fig. 3:**
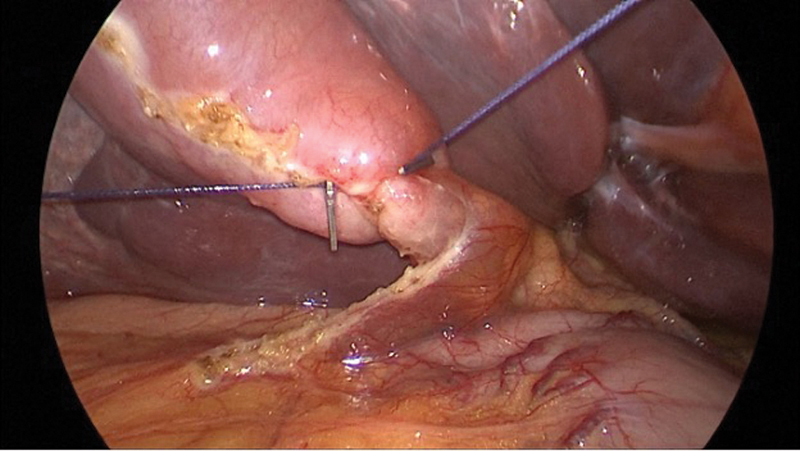
Suture manipulation of gallbladder neck in single-incision laparoscopic cholecystectomy.

A hook is used for dissection, critical view of safety is obtained, cystic duct and artery are clipped and divided, and the gallbladder is dissected in standard fashion. The lateral and medial sutures are cut, inferior port is replaced with an 11-mm trocar, and the gallbladder is retrieved by opening the specimen bag underneath the gallbladder and dropping the gallbladder into it by cutting the vertical suture ( Figs. 1 , 2 , 3 ).

### Statistical Analysis

Chi-square and *t* tests were used to compare baseline patient and clinical characteristics between CLC and m-TLC groups. Univariate and multivariate linear regression were used to determine association of procedure type (CLC vs. m-TLC) with LOS and operative time, while multivariate logistic regression was used to compare the complication rate between CLC and m-TLC. Multivariate analyses were adjusted for age, sex, BMI, ASA class, previous surgical history, and operative indication. All analyses were performed using SAS version 9.4 (SAS Institute Inc., Cary, NC). Results were considered statistically significant at a *p* -value of <0.05 ( Table 1 ).

**Table 1:** Baseline characteristics in patients undergoing marionette transumbilical cholecystectomy and conventional laparoscopic cholecystectomy

Variable	m-TLC ( *n* = 80)	CLC ( *n* = 259)	*p* -Value
Age (y)	38	66	< 0.001
BMI (kg/m ^2^ )	27.3	29	0.01
Female (%)	82.5	54.8	< 0.001
Prior abdominal surgery (%)	30	48.3	0.005
ASA emergency (%)	11.3	20.5	0.069

Abbreviations: ASA, American Society of Anesthesiologists; BMI, body mass index; CLC, conventional laparoscopic cholecystectomy; m-TLC, “marionette technique” for transumbilical laparoscopic cholecystectomy.

## Results

The m-TLC group, as compared with CLC group, comprised patients who were significantly younger (38 vs. 66 years, *p* -value < 0.0001) and more likely to be female (82.5 vs. 54.8%, *p* -value < 0.0001). Further, m-TLC patients had lower BMI (27.3 vs. 29, *p* -value = 0.001), and were less likely to have a history of previous abdominal surgery (30 vs. 48.3%, *p* -value = 0.005).

A statistically significant difference in operative indication was observed between patients undergoing m-TLC and CLC ( Table 2 ). Patients undergoing m-TLC were more likely to have noninflammatory pathology, that is, biliary colic or dyskinesia (80 vs. 45.6%; *p* -value for operative indication <0.001). There was one conversion from m-TLC to CLC; none to open procedure in either group. An IOC was required in 6 patients, which was performed via CLC.

**Table 2:** Operative indication and estimated blood loss in marionette transumbilical cholecystectomy and conventional laparoscopic cholecystectomy

Variable	m-TLC ( *n* = 80)	CLC ( *n* = 259)	*p* -Value
Estimated blood loss (%)			
Minimal (< 30 mL)	92.5	73.7	<0.001
Moderate (30–100 mL)	5.0	23.2	
Severe (> 100 mL)	2.5	3.1	
Operative indication (%)			
Acute cholecystitis	16.3	44.0	< 0.001
Chronic cholecystitis	3.7	10.4	
Biliary colic/dyskinesia	64.0	45.6	

Abbreviations: CLC, conventional laparoscopic cholecystectomy; m-TLC, “marionette technique” for transumbilical laparoscopic cholecystectomy.

Nonadjusted LOS (1 vs. 3 days, *p* -value < 0.0001) and operative time (50 vs. 56 min, *p* -value = 0.007) were significantly lower among patients who underwent m-TLC, as compared with CLC ( Table 3 ). However, in multivariate analyses, procedure type was not a significant predictor of operative time ( *p* -value = 0.131) or LOS ( *p* -value = 0.512).

**Table 3:** Comparison of outcomes between marionette transumbilical cholecystectomy and conventional laparoscopic cholecystectomy (adjusted for age, sex, BMI, ASA class, previous surgical history, and operative indication)

	m-TLC	CLC	*p* -Value
Length of stay (d)	1 (1–3)	3 (2–6)	0.512
Procedure time (min)	50 (43–56)	56 (42–72)	0.131
Complications (%)	2.5	1.9	0.201

Abbreviations: ASA, American Society of Anesthesiologists; BMI, body mass index; CLC, conventional laparoscopic cholecystectomy; m-TLC, “marionette technique” for transumbilical laparoscopic cholecystectomy.

One intraoperative complication (a duodenal serosal tear, which was repaired primarily) was noted in the CLC group. There were no intraoperative complications in the m-TLC group. Postoperative complication rate was 2.5% (retained stones [ *n* = 2]) with m-TLC, and 1.9% (bile leak [ *n* = 2], pneumonia [ *n* = 2], deep venous thrombosis [ *n* = 1]) in patients undergoing CLC. There was no statistically significant difference in the overall complication rate between the two groups (odds ratio: 1.63; 95% confidence interval: 0.02–2.39).

## Discussion

Our study shows that as compared with CLC, m-TLC in a carefully selected population is associated with comparable intra- or perioperative complication rate. Further, unadjusted operative time and LOS were significantly lower in the m-TLC group, while there was no significant difference after adjusting for confounders.

Our findings of lower unadjusted LOS in the m-TLC group are consistent with previous evidence. 9 However, patients undergoing m-TLC are younger, have lower BMI, and are less likely to have inflammatory biliary disease, severe disease, or prior abdominal surgery. In multivariate analyses (when adjusted for these variables), we found no significant difference in LOS between the two groups. Thus, the differences observed in unadjusted analyses are likely a result of confounding. Further, the observed difference in LOS may partly be a result of reduced postoperative pain in m-TLC patients; however, without definitive pain measures, this is difficult to determine. It is also noteworthy that on multivariate analysis, we found no significant difference in operative time between m-TLC and CLC (50 vs. 56 minutes on crude analysis), which is consistent with previous evidence. 4 7 9

There is well-documented randomized evidence that as compared with CLC, SILC is associated with a higher risk of intra- and perioperative complications (relative risk [RR]: 1.41; (95% CI 1.19 -1.68; *p* < 0.001) as well as long-term incidence of hernia. 7 The higher risk of incisional hernias for SILC (RR: 2.97, *p* = 0.005) is likely attributable to the larger 20-mm fascial incision. 12 Complication rates between the two groups in our study did not differ significantly. This is likely a result of excellent retraction and visualization of biliary anatomy using the two intra-abdominal sutures that essentially function as two additional ports, as well as careful patient selection. In the long run, we believe that since m-TLC involves only two fascial incisions (5 and 11 mm), it is likely associated with a lower incisional hernia rate as compared with SILC and CLC, although long-term data are currently lacking.

It is noteworthy that evidence on satisfaction of postoperative cosmetic results overwhelmingly shows superior results with SILC versus CLC. In a systematic review by Lirici et al, 4 10 out of 12 studies reported superior cosmetic results with SILC. While m-TLC employs a different technique, we believe the use of two small incisions which are hidden in the umbilicus leads to cosmetic results and patient satisfaction that are comparable, if not superior, to SILC. It is pertinent to mention here that while m-TLC can be performed in any patient if deemed feasible based on pathology, body habitus, and previous surgical history, its greatest utility is in young patients, especially females, who are most likely to prefer cholecystectomy without any visible residual scar.

Our results showed a significant difference in operative indication between CLC and m-TLC patients; 10.2% of patients with acute cholecystitis underwent m-TLC. Acute cholecystitis has been shown to be associated with a high failure rate for SILC (41%). Similarly, m-TLC to CLC conversion is reported to be as high as 30% in patients with acute cholecystitis. 9 However, our conversion rate in acute cholecystitis patients was only 7.7%, which may be attributable to careful patient selection and the surgeon's technical proficiency in m-TLC.

Evidence suggests that m-TLC is associated with a lower cost of procedure as compared with CLC or SILC (using commercially available SILC ports). 13 The lower procedure cost may significantly reduce the overall cost to the health system since laparoscopic cholecystectomy is one of the most commonly performed surgical procedures.

The limitations of our study include its design, a retrospective review, with its inherent biases. Significant differences between the groups in demographics, operative indication, and severity of disease make comparison difficult. Furthermore, our study was a single-center, single surgeon review which limits its generalizability. Importantly, we did not have objective measures of postoperative pain or need for pain medications, which may be different in the two groups. These limitations demonstrate the need for further high-quality studies comparing m-TLC and CLC.

## Conclusion

For a noncomplicated cholecystectomy, m-TLC is an acceptable option that is associated with comparable operative time and LOS, and no increased risk of intra- or perioperative complications. Furthermore, m-TLC may also offer greater patient satisfaction and superior cosmetic results.
